# Leucistic plumage as a result of progressive greying in a cryptic nocturnal bird

**DOI:** 10.1038/s41598-022-07360-8

**Published:** 2022-03-01

**Authors:** Carlos Camacho, Pedro Sáez-Gómez, Paula Hidalgo-Rodríguez, Julio Rabadán-González, Carlos Molina, Juan José Negro

**Affiliations:** 1 Department of Biological Conservation and Ecosystem Restoration, Instituto Pirenaico de Ecología – CSIC, Av. Nuestra Señora de la Victoria 16, 22700 Jaca, Huesca Spain; 2 Department of Biology, Centre for Animal Movement Research (CAnMove), Lund University, Ecology Building, S lvegatan 37, 223 62 Lund, Sweden; 3 Instituto Multidisciplinar para el Estudio del Medio “Ramon Margalef”, Universidad de Alicante, 03080 Alicante, Spain; 4 Department of Molecular Biology and Biochemical Engineering, University Pablo de Olavide, Carretera Utrera km.1, 41013 Seville, Spain; 5 Observation.org, Fresno 9, 41111 Almensilla, Seville, Spain; 6 Sociedad Española de Ornitología, Centro Ornitológico Francisco Bernis, Paseo Marismeño s.n., 21750 Huelva, Spain; 7 Department of Evolutionary Biology, Estación Biológica de Doñana – CSIC, Av. Américo Vespucio, 26, 41092 Seville, Spain

**Keywords:** Zoology, Ecology, Evolutionary ecology

## Abstract

Leucism, broadly defined as the lack of melanin pigmentation, occurs in many animal species. Most studies on leucism and other colour aberrations are based on opportunistic observations or small cross-sectional samples, thus limiting our ability to produce reliable results and test theoretical predictions. This study combines cross-sectional and longitudinal data collected in 2016–2020 from a population of red-necked nightjars (*Caprimulgus ruficollis*). The goals of the study are (*i*) to investigate sex and age effects on partial leucism, (*ii*) to separate within-subject effects (progressive greying) from between-subject effects (selective disappearance), and (*iii*) to examine differences in body mass, structural size, and life span between leucistic and non-leucistic individuals. The probability of leucism in nightjars increased from juveniles to adults at similar rates in males and females. Our longitudinal analysis and life-span comparisons indicated a minor contribution of selective disappearance to age-related changes in leucism, but rather suggested that the loss of melanin from feathers can be attributed to progressive greying in ageing adults. Body mass and size were consistently smaller (5% and 1.5%, respectively) in leucistic than in non-leucistic nightjars, although the reason for this difference remains unclear. Our study sheds light on the sources and mechanisms of variation in leucism in natural populations and its relationship with important life-history traits, such as life span.

## Introduction

Natural selection theory suggests that phenotypes that deviate from the most common type experience reduced fitness and tend to be eliminated from the population due to increased predation^1,2^ or poor performance resulting from pleiotropic effects^3,4^. Morphological abnormalities are therefore infrequent in natural populations. Current evidence for the action of natural selection on morphological abnormalities is often indirect^5,6^ in part because of the difficulty in obtaining large samples of rare phenotypic traits. Consequently, empirical data to test theoretical predictions and measure the magnitude and direction of predicted effects are virtually non-existent.

Leucism is defined as a series of defects in melanin-producing cells (melanocytes) resulting in the lack of pigmentation in all or parts of the skin (or plumage) of an animal, but not in the soft parts^4,7^. This colour aberration may occur in all vertebrate classes (mammals^8,9^, birds^10,11^, fish^12^, amphibians^13^, reptiles^14^), but it is rare in natural populations. Leucism is thought to result from genetic mutations affecting, for instance, the melanocortin 1 receptor gene^15^. Lack of pigmentation may also result from the so-called progressive greying, that is, a progressive loss or reduction of melanocyte function and the subsequent suppression of melanin formation^16^, although the genetic basis of this process is not fully understood. Unlike congenital leucism, progressive greying starts after the animal reaches a certain age. For simplicity, in this article, the term ‘leucism’ (from the Greek *leukos*, ‘white’) should be interpreted according to its original and all-embracing meaning, as denoting the absence of melanin in the integument, regardless of the underlying process^7^. Lastly, non-genetic (environmental) factors, such as injury^17^ and nutritional constraints on melanization^5^ may also cause loss of pigmentation. Many questions remain about the causes and biological significance of leucism, yet the relative ease of detection of this trait can facilitate data collection and provide useful information to understand the emergence and maintenance of colour aberrations in natural populations^18^.

Leucism is assumed to be disadvantageous in terms of survival due to increased predation risk^8^ or because of pleiotropic effects on other physiological functions^19^. Consequently, as for other morphological abnormalities, the frequency of leucism is expected to remain close to zero, particularly in older age classes^5^. Conversely, the frequency of leucism may increase in older age classes due to a gradual deterioration of physiological function^16^, potentially obscuring or obliterating age-related declines in leucism at the population level. Unbiased estimation of the incidence of leucism therefore requires accounting for ageing effects and changes in the age structure of the population.

Birds are key players in studies on leucism because their conspicuous nature and ease of monitoring facilitate the detection of rare phenotypes. Leucism in birds is defined as the complete or partial absence of eumelanin and pheomelanin in one or more feathers, but not in the eyes, bill or feet, resulting in white patches in all or part of the plumage^4^. Leucism studies of birds are usually based on observations of unmarked animals^10,11,20^ or information from potentially biased sources, such as online photographs^21^ and animals shot for specimens^5,18^. Field studies of individually marked birds, although representing a small proportion of the literature, are generally more reliable and usually include information on the age and sex of individuals^22,23^. Nevertheless, these studies are limited in number and taxonomic scope, being restricted to a handful of predominantly black species (e.g. refs.^5,23,24^; but see Bensch et al*.*^22^). Moreover, due to difficulties in recapturing birds in the field, studies examining age-related changes in leucism^5,23^ are based on cross-sectional comparisons, offering little scope for longitudinal analyses elucidating the role of ageing (senescence) and compositional change across age classes (selection).

This study documents the prevalence of naturally occurring leucism in an intensively studied population of red-necked nightjars (*Caprimulgus ruficollis*), examines sex- and age-related changes in leucism expression, and combines cross-sectional and longitudinal data to investigate the mechanism(s) underlying age-related changes. Nightjars rely on their cryptic plumage and secretive behaviour to escape predation at the nests and roosts^25^. Cryptic plumages, based on melanic patterns, are widespread among nocturnal predatory birds, including nightjars and allies (Order Caprimulgiformes) and owls (Order Strigiformes). These species are mostly active in semidarkness and rest during the day, so they need to either avoid detection by diurnal predators (Caprimulgiformes^26^), or to avert mobbing by small birds during the day (Strigiformes^27^). Cryptic plumage (and secretive behaviour) may thus be deemed critical attributes for these species to enhance survival^25^. Conspicuous plumage markings that are permanently visible at rest, including aberrant white feathers and achromatic plumage ornaments (e.g. white throat badge and wing and tail patches), are expected to be targets of natural selection in cryptic nocturnal species because they can reduce the effectiveness of camouflage to a greater o lesser extent depending on their size and number^26,28^.

Our study population of red-necked nightjars in Doñana National Park (S Spain) provides an excellent opportunity to investigate sex- and age-related changes in leucism expression from a lifetime perspective because (*i*) the sex of individuals, including young, can be accurately determined by plumage characteristics^29^, (*ii*) over 25% of young return to the natal area to breed, and nearly all (80%) do so at age 1^30^, (*iii*) returning individuals can be recaptured over several years due to strong breeding site fidelity^31^, and (*iv*) the annual survival of adults is relatively high (0.64–0.74) and some nightjars can still be recaptured up to 10 + years after the initial ringing date^32^. Moreover, all nightjars are measured for body size and mass, so morphological traits of leucistic individuals can be compared to those of normal-plumaged ones. Our study addresses three questions:

1. **Does the frequency of leucism differ between males and females?** Leucism is often sex-biased, and it is generally assumed to be less common in the sex that experiences the greatest predation risk^19,23^. Leucism in nightjars is expected to be male-biased based on the general assumption that females are under stronger selection than males due to greater susceptibility to predation during nesting^32^.
2. **Does the frequency of leucism differ among age classes?** On the one hand, the differential mortality hypothesis states that leucistic individuals are unlikely to persist long in nature due to the effects of selection against conspicuous plumage markings^5,6^. Under this hypothesis, one would expect an age-related decline in the prevalence of leucism at the population level, reflecting a compositional change across age classes due to the selective disappearance of leucistic individuals. This compositional change should also be reflected in a shorter life span in leucistic individuals compared to normal-plumaged ones. On the other hand, the progressive greying hypothesis states that leucism increases in the older segments of the population as a result of the partial or total loss of melanin from feathers^4,33^. Under the progressive greying hypothesis, one would expect an age-related increase in the prevalence of leucism at the population level due to the effects of intra-individual variability in leucism expression.
3. **Do leucistic individuals differ in skeletal size or body mass from normally coloured individuals? **Leucistic individuals have been found to be smaller than average, apparently because of nutritional constraints on plumage melanization –and therefore skeletal development– during the nestling stage^5^. Environmental effects on leucism expression in nightjars, if any, are expected to result in smaller body size and body mass compared to normal-coloured individuals.

## Results

**Figure 1:**
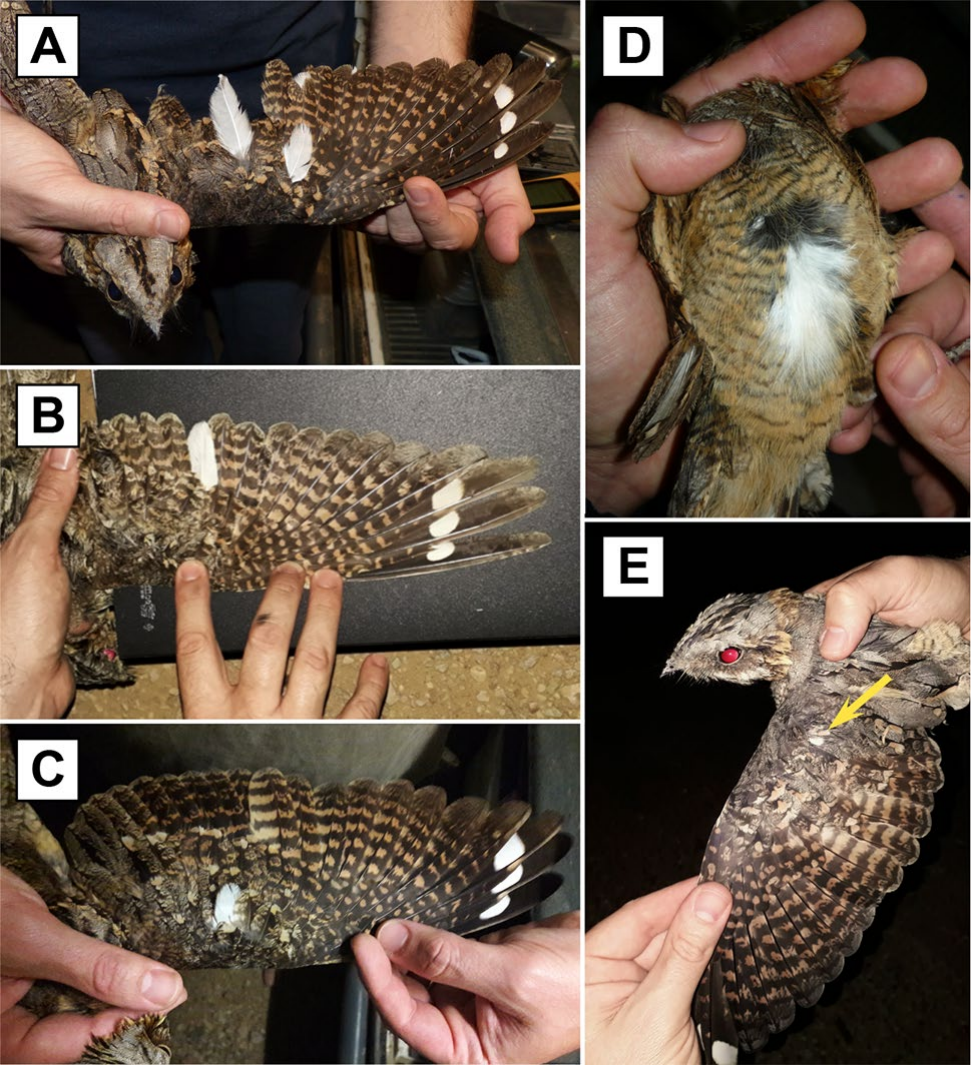
Leucism in red-necked nightjars. Example images illustrating variation in the number and location of unpigmented (leucistic) feathers in adult birds: one great cover, one secondary, and the tip of the outermost secondary (**a**), one secondary only (**b**), one medium covert (**c**), one contour feather (**d**), and one lesser covert (**e**). Photographs by Carlos Camacho.

Our data set included 1,092 observations of 572 adults (*n* = 154 ringed as juveniles and 418 ringed as adults) from 12 cohorts (2009–2020), and 774 observations of 508 juveniles from 5 cohorts (2016–2020), spanning an age range of 0–8 years. Out of the 572 adults, 7 males and 11 females showed unpigmented feathers (Tables 1 and 2). The affected feathers, far from being randomly distributed, typically included lesser coverts (15%), median coverts (55%), and greater coverts (20%), although leucism also affected one secondary in two birds (Fig. 1A, B), and one body contour feather in one of the birds captured before the study (Fig. 1D). Only one bird (a female) presented more than one unpigmented feather (Fig. 1A, Table 1). The annual and sex-related frequency of leucism among adults ranged from 0% to 5.7% depending on the year and sex (Table 2). Not a single case of leucism was observed among the 508 juveniles examined over the five years of study.

**Table 1:** Red-necked nightjars showing partial leucism at some time during the 5-year (2016–2020) study (N = 18) or earlier (N = 2). Details on sex, period from first to last capture, life span, cohort (only for birds ringed as juveniles), number of leucistic (white) feathers and feather tract(s) affected are shown.

ID	Sex	Tracking period	Life span	Cohort	Expression age	No.feathers	Tract(s)
3368884	M	2011–2016	5	2011	??	1	LC
3037273	F	2012–2018	6	2012	1	1	GC
3396914	M	2013–2017	4	2013	??	1	MC
3396924	M	2013–2015	2	2013	1 +	1	MC
1B09505	F	2015–2020	6	2015	2	1	MC
1B09574	F	2015–2020	5	2015	2	1	MC
1B09597	M	2015–2018	4	2015	??	1	MC
1B28187	M	2017–2019	2	2017	1	1	MC
1B24337	F	2017–2020	3	2017	??	1	SS
1B47008	F	2018–2020	3	2018	2	1	LC
1B47089	F	2019–2020	2	2018	2	1	LC
1B09596	F	2015–2019	5 +		??	1	MC
1B28425	F	2018–2020	3 +		2 +	1	MC
1B23574	F	2016–2019	4 +		??	1	MC
1B23710	M	2016–2019	4 +		3 +	1	GC
3222399	M	2010–2017	8 +		??	1	MC
3260995	F	2011–2017	7 +		??	1	MC
3288680	M	2013–2019	7 +		??	1	GC
1B23782	F	2016–2016	1 +		??	3	GC + SS
3185551	F	2009–2014	6 +		??	1	BC

For birds ringed as adults, the minimum estimated life span and age of leucism expression is presented. M = male, F = female, LS = lesser covert, MC = medium covert, GC = great covert, SS = secondary, BC = body contour.

**Table 2:** Frequency of leucistic adult (≥ 1 year-old) red-necked nightjars in Doñana during 2016–2020.

year	Number leucistic		Total number		Frequency (%)	
	males	females	males	females	males	females
2016	2	2	64	107	3.13	1.87
2017	2	4	74	98	2.70	4.08
2018	2	0	75	108	2.67	0.00
2019	3	3	78	72	3.85	4.19
2020	0	5	43	88	0.00	5.68
2016–2020	7	11	230	342	3.04	3.22

### Cross-sectional sex and age effects

**Figure 2:**
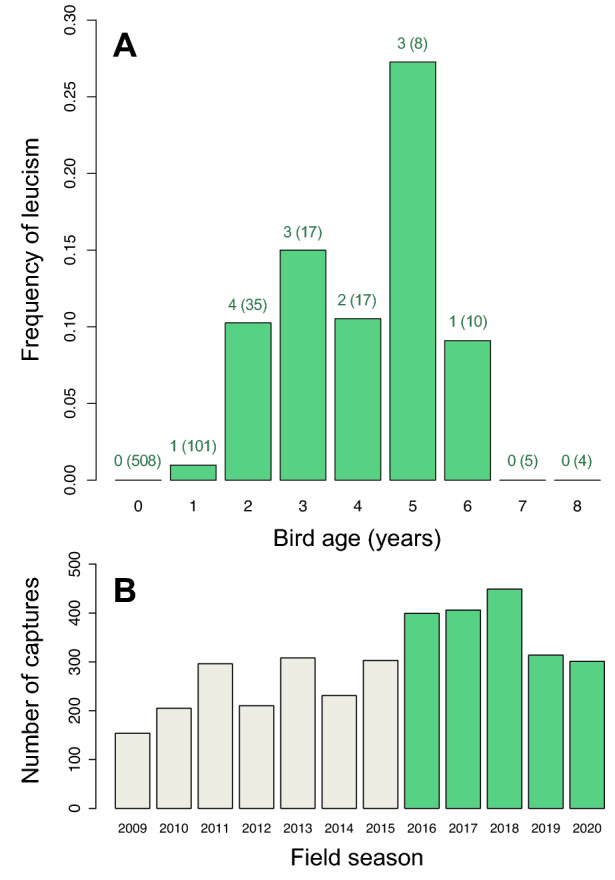
Changes in the frequency of leucism across age classes and annual numbers of captures. (**a**) Frequency of leucism in red-necked nightjars first captured as juveniles and aged 0–8 years. Numbers above bars are the number of leucistic birds in each age category and sample sizes in parenthesis. Note that some individuals appear in more than one category due to repeated trapping in different years. (**b**) Number of captures (including intra-annual recaptures) during the study period (2016–2020, green) and prior to study initiation (2009–2015, grey).

The frequencies of leucism for all years combined amounted to 3.0% and 3.2% for adult males and adult females, respectively (Table 2) and increased from the extremes (0 and 8 years) to the middle (3–5 years) of the age distribution (Fig. 2A). The predicted probability that a trapped nightjar would have a white feather (i.e., the probability of leucism) was low for all ages and sexes (< 0.015%) and did not differ significantly between sexes regardless of age (Table 3). Sex accounted for a negligible proportion of the variance in the probability of leucism in the population (*R*^2^_m_ = 0.0001). By contrast, age explained about 8% of the variance in the probability of leucism (*R*^2^_m_ = 0.0815). Specifically, the probability of leucism (estimated from the inverse logit of the predicted log-odds in Table 2) increased by eight orders of magnitude from the first year of life (~ 10^–13^) to age 4 years (~ 10^–5^) and then decreased to almost zero (~ 10^–9^–10^–12^) at age 7–8 years (Table 1).

**Table 3:** Results of the mixed models analysing (a) sex and age effects on the probability of leucism (coefficients on the log-odds scale), and (b, c) body-size differences between leucistic and non-leucistic individuals.

	Estimate	SE	z	P
**(a) Probability of leucism**				
Intercept	− 28.536	9.594	− 2.974	0.0029
Sex (female)	− 0.033	8.834	− 0.004	0.8545
Age	9.592	3.375	2.842	< 0.0001
Age^2^	− 1.196	0.432	− 2.770	< 0.0001
Sex × Age	0.143	2.160	0.066	0.9482
**(b) Skeletal size (sternum length)**				
Intercept	32.796	0.160	204.430	< 0.0001
Leucism (yes)	− 0.459	0.202	− 2.270	0.0236
Sex (female)	− 0.329	0.100	− 3.280	0.0011
Age	0.141	0.029	4.900	< 0.0001
**(c) Body mass**				
Intercept	84.003	1.191	70.530	< 0.0001
Leucism (yes)	− 4.082	1.990	− 2.050	0.0405
Sex (female)	0.590	0.628	0.940	0.3472
Age	1.994	0.249	8.000	< 0.0001
Gizzard size	3.351	0.248	13.520	< 0.0001

Only birds of exactly known age (i.e., ringed as juveniles) are considered in these analyses. Number of observations: 1054 (a), 999 (b), and 1037 (c). Number of individuals: 563 (a), 561 (b), and 559 (c). Number of cohorts: 12 in all the three models.

The age of first expression for 6 individuals ringed as juveniles averaged 1.8 ± 0.4 (SD) years (Table 1). Only one of these birds expressed a white feather at age 1 (second-calendar year), although moult data collected from this individual on three occasions (May, August and September) during the breeding season 2017 revealed that this feather emerged during the first post-nuptial moult in late summer. Examination of leucism expression in individuals recaptured in more than one year (all but one) revealed that they had the same white feathers in all years they were examined, indicating that leucism expression in nightjars is not a transient reversible phenomenon.

### Effect of selective disappearance and ageing on age-related variation in leucism

The within-subject centering analysis showed a significant effect of intra-individual variability in leucism, and a non-significant antagonistic effect of inter-individual variability (Table 4a), these effects being statistically different from each other (Table 4b). Taken together, these results indicate that the contribution of intra-individual (ontogenetic) changes to overall age-related variation in leucism is large enough to counteract the (negligible) effects of selective disappearance and thus explain the observed population-level patterns.

**Table 4:** Results of the mixed models analysing age-related variation in leucism within and among individuals.

	Estimate	SE	z	P
**(a) Main model**				
Intercept	− 35.088	10.243	− 3.426	< 0.0001
Within-subject effect	7.700	2.527	3.047	0.0023
Between-subject effect	− 1.971	2.960	− 0.666	0.5054
**(b) Reformulated model**				
Intercept	− 35.088	10.243	− 3.426	< 0.0001
Age	20.584	6.755	3.047	0.0023
Between-subject effect	− 21.061	7.774	− 2.709	0.0068

Only birds ringed as juveniles and one observation per bird and year are included in this analysis. Note that, in the reformulated model (b), the between-subject effect represents the difference between the between-subject and the within-subject effects in the main model (a) (see main text for details). These models did not include sex based on the results of the cross-sectional analysis presented in Table 3a. Number of observations: 719. Number of individuals: 563. Number of cohorts: 12.

### Longevity and morphology of leucistic vs. non-leucistic nightjars

The mean life span (± SD) of leucistic nightjars was 3.5 ± 1.6 years (N = 11), compared to 2 ± 1.6 years for their normal-plumaged counterparts (N = 147), although this difference was not statistically significant (permutation *t*-test, *t* = 2.95, *d.f.* = 156, *P* = 0.154). Body mass and skeletal (sternum) size were consistently smaller in leucistic adults compared to non-leucistic adults (ca. 5% and 1.5% difference for body mass and sternum length, respectively).

## Discussion

Leucism is a common condition in vertebrates, especially in birds, and reports of leucistic forms are plentiful. Despite this attention, the condition has usually been treated as a curiosity rather than as a measurable trait and, although there are studies looking at age- and sex-related changes in leucism^5,18,22,23^, they are scarce and focused on differences at the population level. Consequently, the connection –or lack thereof– between population-level and individual-level variability in leucism remains to be established. Our study investigated age and sex effects on the probability of leucism in an intensively studied population of red-necked nightjars and extended these analyses by quantifying within- and between-individuals variability in leucism expression across ages. In addition, we examined life span and body size differences between leucistic and non-leucistic individuals. Our study revealed that: (*i*) there is considerable annual variation in the frequency of leucism, ranging from 0 to more than 5:10^2^ depending on the year and sex group; (*ii*) the probability of leucism is not affected by sex, but increases with age at a comparable rate in males and females; (*iii*) this increase is due to an ontogenetic shift in leucism expression; (*iv*) there is no sign of compositional change across ages, as may be expected from leucism-biased mortality or reduced life span of leucistic birds; (*v*) normal birds are significantly larger in size than leucistic birds.

The frequency of leucism in this red-necked nightjar population (3:10^2^) is among the highest yet recorded in natural bird populations (range: 7:10^6^ to 1:10^2^; refs.^10,11,22^; but see refs.^23,34^). No estimates for nocturnal or other cryptic birds exist in the literature, making direct comparisons to our results difficult. Nevertheless, the frequency of leucism in nightjars is close to the ones recorded for nocturnal mammals (bats)^9^. The smaller prevalence reported in most other bird studies could reflect a detection bias resulting from the use of indirect approaches, such as citizen science data and online search tools^21^. But, in any case, the direction of this bias should act to increase, rather than decrease, the actual frequencies of leucism, because rare plumage patterns tend to be overrepresented in non-systematic surveys and image repositories^21^. Our estimates are unlikely to be confounded by such methodological bias, since they are based on the close examination of individuals captured during a systematic field-based survey.

Male and female nightjars exhibited leucism at comparable rates. This finding does not support the prediction that the presumably greater susceptibility of nesting females to predation generates a male bias in leucism. Female nightjars incubate the eggs and care for the chicks during the day, but it is the male that takes over these duties during much of the night^35^. Thus, predation risk for breeding males and females might not be as different as generally assumed for nightjars – but see Forero et al*.*^32^ for a discussion of rainfall effects on sex-dependent predation risk. Estimates of the frequency of leucism by sex are lacking for other cryptic species, yet the sex ratios reported in studies of non-cryptic species range from male-biased (*Turdus merula*)^23^ to female-biased (*Acrocephalus arundinaceus*)^22^ or equal (*Corvus corone*, *Hirundo rustica)*^5,24^*.* It therefore seems that sex effects on leucism in birds may be species-specific or even vary among populations of the same species^22,36^.

The predicted probability of leucism in this nightjar population increased markedly from the juvenile to the adult stage; in fact, leucism did not occur in juvenile plumage. Based on cross-sectional data from urban and rural blackbird (*Turdus merula)* populations, Izquierdo et al*.*^23^ also found an age-related increase in leucism and interpreted this increase as preliminary support for the progressive greying hypothesis. Our study confirms and extends this finding through a longitudinal individual-level approach demonstrating an ontogenetic shift in leucism expression. Our data furthermore indicate a negligible effect of selective disappearance, suggesting that age differences in the prevalence of leucism in this nightjar population are (almost) entirely the result of changes in the normal plumage pattern due to the progressive loss of melanin.

Under the progressive greying hypothesis, the number of white feathers may be expected to increase after every moult^23,37^, as is the case for the grey hair of humans and other mammals^38^. Only one of all nightjars examined in this study presented more than one white feather, suggesting that progressive greying in this cryptic species implies a qualitative transition. Cross-sectional analyses actually indicated that the probability of leucism in nightjars decreased by several orders of magnitude in extreme old age. This pattern might at first sight seem to suggest that either partial leucism is reversible or that leucistic individuals progressively disappear from the population, but there is evidence that strongly argues against these possibilities. First, consistency over time in the expression of the same white feathers in repeatedly recaptured nightjars supports the notion that leucism is not a reversible phenomenon^18^. Second, the simultaneous analysis of cross-sectional and longitudinal data suggested negligible or small effects of selective disappearance. Mortality gradually reduces the numbers of animals from cohorts marked as young, so that the number of individuals available for analysis in the older age classes is often limited. This reduction in the sample limits our ability to detect and interpret senescence patterns^39^. Our sample of very old individuals (7 + years) is small (1.2% of the data; Fig. 2A) and probably too small to capture naturally rare forms like leucism; therefore, it seems most likely that, in contrast to juveniles, the estimated decrease in the occurrence and predicted probability of leucism at the upper tail of the age distribution is a representativeness bias affecting the oldest segment of the population, rather than a true biological effect. Larger longitudinal data sets are needed to check the validity of this assumption.

Leucistic individuals are generally assumed to be less likely to persist in nature than normally coloured individuals, resulting in an age-related decrease in the mean probability of leucism. Our results do not support this assumption, since all the juveniles examined in this study lacked white feathers, these being detected after the first postnuptial moult at the earliest. Moreover, the long life span of leucistic nightjars and the minor contribution of population-level processes to age-related changes in leucism also argue against the selective removal of leucistic individuals from the population. Usually, the life span of leucistic birds is shorter than that of normal-plumaged birds, although in many cases this difference may be explained by the artificial effects of selective hunting^5,40^. Our results suggest that neither natural nor artificial sources of mortality appear to pose a major threat to leucistic nightjars. However, it should be noted that the unpigmented patches observed in nightjars are almost imperceptible at rest and might actually reduce detection through disruption^41^. Leucism generally affected just one single median or lesser covert (< 1% of the visible plumage of an individual), in contrast to the cases of extensive or complete leucism reported in other birds^10,11^. Completely leucistic individuals may find it difficult to evade detection by predators^25^, yet the small number and size of white feathers exhibited by most nightjars are probably not conspicuous enough to impair camouflage through background matching and reduce survival^6^. Our study provides little support for the hypothesis that partial leucism is an adverse condition for nightjars, although it is still possible that natural selection acts to limit the number and extension of white patches through the removal of more conspicuous variants.

Our data cannot be used to determine the proximate mechanism(s) responsible for progressive greying, though some inferences can be made. For instance, some studies suggest that the frequency of leucism may increase in small, isolated populations due to the effects of inbreeding^8,22,42^, but neither population-size nor dispersal limitations appear to be strong enough to cause significant inbreeding in this nightjar population^30,31^. Environmental factors could also influence the appearance of leucistic forms, regardless of their genetic basis. Nutritional deficiencies due to poor diet may disrupt melanin pigmentation at feather development and thus promote the appearance of partial leucism^5^. Leucistic nightjars did not reach the size of individuals of normal plumage colouration, suggesting that they may have experienced poor nutritional conditions during development. Nonetheless, the pheomelanins that produce the plumage pattern of nightjars are not acquired through the diet, but synthesized by the birds themselves^43^. Nutritional constraints on plumage melanization can still operate if the environmental availability of pheomelanin precursors –mainly cysteine– is restricted during feather development^16,44^, but then feather depigmentation should occur not only in adults, but also in juveniles^5^. Furthermore, the consistent expression of white feathers in the same position over successive moults argues against this possibility.

Exposure to natural or anthropogenic factors promoting oxidative stress, such as extreme temperatures, radiation, and certain pro-oxidative pollutants, might alter the pigmentation process and precipitate progressive greying^16^. For example, the effect of radiation on metabolic function in barns swallows (*Hirundo rustica*) breeding around Chernobyl (Ukraine) after the nuclear catastrophe induced an increase in the frequency of partial leucism compared to the situation before the catastrophe^19,24^. Our study population is located near greenhouse crops of berries that are visited by nightjars on a daily basis for nocturnal foraging^30^. Exposure to the pesticides used in these crops^45^ could expose nightjars to increased oxidative stress and thus promote the appearance of leucism, as has been proposed to explain the greater incidence of leucism in urban populations of blackbirds (*Turdus merula*) compared to rural ones^23,46^. Finally, it would be worth considering the possibility that leucism, so far considered a genetic condition, had in fact an epigenetic origin due to exposure to environmental stressors early in the ontogeny^47^. Under the expected scenarios of global change, experimental studies based on longitudinal individual-based data are needed to elucidate the (anthropogenic) factors influencing the occurrence of morphological and colour aberrations in animals.

To sum up, our results demonstrate an ontogenetic shift in the expression of partial leucism in male and female red-necked nightjars due to progressive greying. This shift is not confounded by the selective disappearance of leucistic individuals, suggesting that partial leucism in nightjars is, unlike in non-cryptic species studied so far, not conspicuous enough to increase predation risk. Our study provides important information for a better understanding of the sources of variation in leucism in natural populations and its effects on important life-history traits, such as life span. The question as to why leucistic birds are smaller than the rest of the population is intriguing and deserves further investigation.

## Methods

### Study system

Data on the prevalence of leucism were collected between 2016 and 2020 as part of a long-term study of red-necked nightjars in Doñana National Park^31,48,49^. The study area (37°7’N, 6°33’ W) consists of a 2000-ha mosaic of sparse Mediterranean shrublands, cattle grasslands, and pine tree plantations. Human access to this area is limited, as it is granted only for scientific research and occasional small-game hunting and livestock management (see Camacho et al*.*^35^ for a detailed description of the study area).

The red-necked nightjar (hereafter ‘nightjar’) is a trans-Saharan migratory species that breeds in Mediterranean areas of the Iberian Peninsula and North Africa^50^. Most individuals arrive in the study area during May and return to their wintering grounds in West Africa between September and October^49^. Nightjars produce one or two clutches of 1–2 eggs per breeding season (May–August). Chicks fledge at 18–22 days of age and, although they are able to forage for themselves, both parents continue to care for them until the 2^nd^–3^rd^ week post-fledging^49^.

Moult strategies in nightjars are complex, but general differences among age classes have been described. Birds in their first calendar year may undergo a post-juvenile partial moult before migration, including body contour feathers and, exceptionally also, 1–2 inner secondaries and greater coverts, but not median or lesser coverts. Birds in their second calendar year replace a small number of flight feathers (e.g., the central pair of rectrices, 1–2 outermost secondaries, 1–2 innermost primaries, and the corresponding upper coverts) before returning to their breeding grounds for the first time at age 1 year^30,51^. First-time breeders therefore display two generations of feathers. Upon completion of breeding in late summer, all nightjars undergo an extensive post-nuptial moult of up to 10–15 flight feathers^49^ and replace the rest of feathers during their second winter in Africa^51^. From that moment on, the moulting sequence is the same as that described for first-time breeders.

### Field procedures

From early April to late October, we conducted nocturnal surveys for red-necked nightjars along a 24 km road transect. Nightjars are attracted to roads during the night because they use their bare surfaces as a foraging platform to locate and capture passing insects^52^, and also to reduce thermoregulation costs on cold nights, take grit for mechanical digestion, and facilitate escaping from approaching predators^35,49^. Nocturnal surveys started one hour after sunset and continued until we covered the 24-km transect by driving a car at slow speed (30 km h^-1^). Nightjars found on roads were captured using a torch and a 80-cm diameter butterfly net^53^ and were marked with numbered metal rings (if not already ringed). The annual number of captures during this study ranged from 301 to 449 (mean = 374, SD = 63.6), amounting to a total of 1,869 captures (Fig. 2B).

Birds first captured as juveniles of the year (described as age 0) were of known age. Those first captured as adults in their second or later calendar years were assigned an age of 1 year or a minimum age of 2 years, respectively, according to plumage characteristics^29,49^. Natal philopatry of nightjars is strong^30^ (recruitment rate = 26.4%, N = 655 marked juveniles from the 2009–2017 cohorts) and, as a result, the age of 152 (27%) adults examined during 2016–2020 could be precisely determined from ringing data. Both juveniles and adults were sexed based on the size of the white patches on the tail and wings^32^ and measured for sternum (keel) length (± 0.01 mm), a predictor of skeletal size in birds^54^ and body mass (± 0.1 g). Body mass is affected by the amount of food ingested. This effect is particularly important in caprimulgids due to their large stomach capacity^52^. External examination of the degree of fullness of the muscular stomach of nightjars (gizzard) provides a simple method to estimate the amount of food ingested and therefore correct body-mass measurements. For each bird, we scored gizzard size as empty, ¼, ½, ¾ or full based on the criteria of Jackson^52^.

For each individual, we noted the presence or absence of (abnormal) white feathers (Fig. 1) and recorded their number and location (feather tract). The naming and numbering of the feather tracts follows Svensson^55^. Birds in active moult could have lost unpigmented feathers prior to capture, resulting in an underestimation of their prevalence. To account for this potential bias, we recorded the state of moult of each individual as the total number of growing primaries and rectrices^49^. Exploratory analyses revealed no effect of state of moult on the probability of leucism, so the effect of feather loss was not considered further in the analyses.

Nightjars tend to return to the same breeding site year after year^31^ and are often captured over several years (mean interannual recapture rate of adults = 0.60 ± 0.24 (SD), N = 39 individuals tracked over 2–7 more years after recruitment). Longitudinal data from repeated observations of birds ringed as juveniles were used to determine the age of first expression of white feathers, to assess plasticity in leucism expression throughout life, and to estimate the life span of leucistic and normal-coloured birds. Life span was defined as the age of birds during their last breeding season at our study site. Life span estimates are presented as minimum longevity, since some birds may still be alive at the end of the study. Birds first caught before the onset of the present study (2016) or those captured intermittently during the study could have presented white feathers earlier than recorded, so the age of expression in these birds could not be determined. Non-systematic records of white feathers in birds captured before 2016 nevertheless provided useful information about the age of first expression for three adults. To maximize the sample size, in the analysis of annual frequencies by sex we considered both birds ringed as juveniles and those ringed as adults.

### Data analyses

#### Cross-sectional patterns

To investigate sex and age effects on the probability of leucism in nightjars, we ran a generalized linear mixed model (GLMM, binomial distribution and a logit link function) using leucism (0 = no leucistic feathers, 1 = at least one leucistic feather) as the dependent variable. Only birds of precisely known age were considered in this analysis. Four out of the 1,869 records (0.2%) were excluded due to missing data. The model included sex (class variable), age (continuous covariate), quadratic age, and the interaction between sex and age as explanatory variables. Bird identity (ring number) and year of birth were included as random intercepts to account for repeated measurements of the same individuals and cohort effects, respectively. The addition of the age-specific random slope to the model increased the percentage of variance explained by only 0.3% and its effect did not reach statistical significance (LRT: Chisq = 4.99, *d.f.* = 2, *P* = 0.083; see details on the tests used at the end of the ‘Data analysis’ section). For simplicity, the model included a random intercept for individual only.

#### Decomposing changes in average leucism expression across ages

To separate contributions of within-individual changes and between-individual selective disappearance to the age pattern at the population level we used the within-subject centering approach^56^. This method enabled us to partition the total variance of leucism into two independent components: the between-subject component of variance, calculated as the average age of each individual (*x̄*_*j*_), and the within-subject component, calculated by subtracting these average values from each observation value (*x*_*ij*_– *x̄*_*j*_)^56^. To statistically assess the contribution of each component to age-related variation in leucism, we fitted the *x̄*_*j*_ and *x*_*ij*_– *x̄*_*j*_ terms into a GLMM with leucism (0/1) as the response variable. The model included the random effect of year of birth to account for cohort effects, and also the random intercept and age-specific slope of each individual to account for the non-independence of data points and to minimize the probability of obtaining overconfident estimates^57^. To determine whether the within-individual and between-individual components of variation differed statistically from each other, we reformulated the model including the original age predictor (*x*_*ij*_) and the individual’s mean age term (between-subject component), but not the centred age predictor (*x̄*_*j*_). The individual’s mean age term (*x*_*ij*_– *x̄*_*j*_) in this model represents the difference between the between-subject and the within-subject effects in the previous model and is expected to be non-significant when there is no difference between them (see formula 3 in van de Pol and Wright^56^).

#### Longevity analysis

Leucistic nightjars are a non-random subset of the population (≥ 1-year-old adults) due to age effects on leucism expression (see ‘Results’). Thus, to facilitate comparison of the life span of leucistic and normal-plumaged birds, the analysis of this trait only included individuals that reached the adult stage to avoid underestimating the life span of normal-plumaged birds due to the inclusion of non-recruiting juveniles (69%, N = 614 ringed juveniles). We compared the mean life span of leucistic *vs.* normal-plumaged individuals with a permutation *t*-test (9,999 permutations) to account for the unbalanced nature of the data (N = 11 leucistic *vs.* 157 normal-plumaged) and non-normal distribution (Shapiro–Wilk normality test, *W* = 0.729, *P* < 0.001). To calculate the *p*-value of the *t*-test, we compared the observed difference between means to the frequency distribution of the simulated values.

#### Morphometric differences between leucistic and non-leucistic individuals

To test for morphometric differences between leucistic and normally coloured nightjars, we fitted separate linear mixed models (LMM, normal error distribution and identity link function) to the sternum length and body mass data, considered to be good predictors of general body size in birds^54,58^. These models included leucism (0/1) as the explanatory variable, and sex and a fixed effect to control for differences in size between males and females and among age groups. The LMM fitted to body mass data also included gizzard score to account for the influence of food consumption on body mass upon capture. Bird identity and year of birth were also used as controlling random variables, as explained above.

GLMMs and LMMs were fitted using the ‘glmmTMB’ package^59^ of the R environment, version 4.0.0 (http://cran.r-project.org/). Binary data are often zero-inflated, but the results of residual diagnostic tests (e.g. quantile–quantile plots) in the DHARMa package^60^ indicated that the GLMMs provided a good fit to the data even in the presence of excess zeros. To compute *p*-values for individual predictors included in the models, we compared full models containing all variables to reduced models excluding the predictor of interest using a likelihood ratio chi-square test (LRT). To estimate the proportion of variance explained by both random and fixed effects and the fixed effects only, we calculated the conditional and marginal *R*^2^ values (*R*^2^_c_ and *R*^2^_m_, respectively) according to Nakagawa & Schielzeth^61^, using the r.squaredGLMM function of the ‘MuMIn’ package^62^.

### Experiments on live vertebrates

The authors declare that all procedures have been approved by the ‘Dirección General de Medio Natural, Biodiversidad y Espacios Protegidos’ (regional authority) through the permits 2,016,107,300,002,288/FQH/MDCG (years 2016–2019) and 202,010,730,000,647/FQH/MDCG (year 2020). This study did not involve threatened or endangered species and was carried out in accordance with national and international guidelines for care and use of animals.

## Data Availability

The data supporting the results of this study are available from the repository of the Spanish National Research Council (CSIC) and can be accessed at: http://hdl.handle.net/10261/253208.

## References

[1] Rutz C (2012). Predator fitness increases with selectivity for odd prey. Curr. Biol..

[2] Santos CD (2015). Personality and morphological traits affect pigeon survival from raptor attacks. Sci. Rep..

[3] Brown MB, Wells E (2020). Skeletal dysplasia-like syndromes in wild giraffe. BMC Res. Notes.

[4] van Grouw H (2013). What colour is that bird? The causes and recognition of common colour aberrations in birds. Br. Birds.

[5] Slagsvold T, Rofstad G, Sandvik J (1988). Partial albinism and natural selection in the hooded crow *Corvus corone cornix*. J. Zool..

[6] Stevens M (2013). Revealed by conspicuousness: distractive markings reduce camouflage. Behav. Ecol..

[7] van Grouw H (2021). What’s in a name? Nomenclature for colour aberrations in birds reviewed. Bull. Br. Ornithol. Club.

[8] Parsons GJ, Bonderup-Nielsen S (1995). Partial albinism in an island population of Meadow Voles, *Microtus pennsylvanicus*, from Nova Scotia. Can. Field-Nat..

[9] Reis, A. da S., Zampaulo, R. de A. & Talamoni, S. A. Frequency of leucism in a colony of *Anoura geoffroyi* (Chiroptera: Phyllostomidae) roosting in a ferruginous cave in Brazil. *Biota Neotropica* **19**(3): e20180676. 10.1590/1676-0611-BN-2018-0676 (2019).

[10] Jehl JR (1985). Leucism in Eared Grebes in western north America. Condor.

[11] Forrest S, Naveen R (2000). Prevalence of leucism in Pygocelid penguins of the Antarctic peninsula. Waterbirds.

[12] González-Ortegón E, Drake P, Quigley DTG, Cuesta JA (2020). Leucism in the European sardine *Sardina pilchardus* (Clupeidae). Ecol. Indic..

[13] David BZ (2021). First report of partial leucism in the poison frog *Epipedobates anthonyi* (Anura: Dendrobatidae) in El Oro, Ecuador. Neotrop. Biodivers..

[14] Krecsák L (2008). Albinism and leucism among European Viperinae: a review. Russ. J. Herpetol..

[15] Ritland K, Newton C, Marshall HD (2001). Inheritance and population structure of the white-phased “Kermode” black bear. Curr. Biol..

[16] Galván I, Bijlsma RG, Negro JJ, Jarén M, Garrido-Fernández J (2010). Environmental constraints for plumage melanization in the northern goshawk *Accipiter gentilis*. J. Avian Biol..

[17] Pijpe, A., Gardien, K. L. M., Meijeren-Hoogendoorn, R. E. van, Middelkoop, E. & Zuijlen, P. P. M. van. Scar Symptoms: Pigmentation Disorders in *Textbook On Scar Management* (eds. Téot, L., Mustoe, T. A., Middelkoop, E. & Gauglitz, G. G.) 109–115 (Springer, 2020). 36351145

[18] Edelaar P (2011). Apparent selective advantage of leucism in a coastal population of Southern caracaras (Falconidae). Evol. Ecol. Res..

[19] Ellegren H, Lindgren G, Primmer CR, Møller AP (1997). Fitness loss and germline mutations in barn swallows breeding in Chernobyl. Nature.

[20] Benítez-López A, García-Egea I (2015). First record of an aberrantly colored Pin-tailed Sandgrouse (*Pterocles alchata*). Wilson J. Ornithol..

[21] Zbyryt A, Mikula P, Ciach M, Morelli F, Tryjanowski P (2020). A large-scale survey of bird plumage colour aberrations reveals a collection bias in Internet-mined photographs. Ibis.

[22] Bensch S, Hansson B, Hasselquist D, Nielsen B (2000). Partial albinism in a semi-isolated population of Great Reed Warblers. Hereditas.

[23] Izquierdo L (2018). Factors associated with leucism in the common blackbird *Turdus merula*. J. Avian Biol..

[24] Møller AP, Mousseau TA (2001). Albinism and phenotype of barn swallows (*Hirundo rustica*) from Chernobyl. Evolution.

[25] Troscianko J, Wilson-Aggarwal J, Stevens M, Spottiswoode CN (2016). Camouflage predicts survival in ground-nesting birds. Sci. Rep..

[26] Aragonés J, Arias de Reyna L, Recuerda P (1999). Visual communication and sexual selection in a nocturnal bird species, *Caprimulgus ruficollis*, a balance between crypsis and conspicuousness. Wilson Bull..

[27] Negro JJ, Bortolotti GR, Sarasola JH (2007). Deceptive plumage signals in birds: manipulation of predators or prey?. Biol. J. Linn. Soc..

[28] Brooke, M. de L. Unexplained recurrent features of the plumage of birds. *Ibis* **152**, 845–847 (2010).

[29] Forero MG, Tella JL, García L (1995). Age related evolution of sexual dimorphism in the Red-necked Nightjar *Caprimulgus ruficollis*. J. Ornithol..

[30] Camacho C (2014). Early age at first breeding and high natal philopatry in the Red-necked Nightjar *Caprimulgus ruficollis*. Ibis.

[31] Camacho C (2016). The road to opportunities: landscape change promotes body-size divergence in a highly mobile species. Curr. Zool..

[32] Forero MG, Tella JL, Oro D (2001). Annual survival rates of adult Red-necked Nightjars *Caprimulgus ruficollis*. Ibis.

[33] Henner J (2002). Genetic mapping of the (G)-locus responsible for the coat color phenotype “Progressive Greying with Age” in horses (*Equus caballus*). Mamm. Genome.

[34] Edson JM (1928). An epidemic of albinism. Auk.

[35] Camacho C, Palacios S, Sáez P, Sánchez S, Potti J (2014). Human-induced changes in landscape configuration influence individual movement routines: lessons from a versatile, highly mobile species. PLoS ONE.

[36] Enders F, Post W (1971). White-spotting in the genus Ammospiza and other grassland sparrows. Bird-Band..

[37] Sage BL (1962). Albinism and melanism in birds. Br. Birds.

[38] O’Sullivan JDB (2021). The biology of human hair greying. Biol. Rev..

[39] Nichols JD, Hines JE, Blums P (1997). Tests for senescent decline in annual survival probabilities of common pochards, *Aythya ferina*. Ecology.

[40] Owen M, Skimmings P (1992). The occurrence and performance of leucistic Barnacle Geese *Branta leucopsis*. Ibis.

[41] Mulder T, Campbell CJ, Ruxton GD (2021). Evaluation of disruptive camouflage of avian cup-nests. Ibis.

[42] Holyoak D (1978). Variable albinism of the flight feathers as an adaptation for recognition of individual birds in some Polynesian populations of Acrocephalus warblers. Ardea.

[43] Griffith SC, Parker TH, Olson VA (2006). Melanin- versus carotenoid-based sexual signals: is the difference really so black and red?. Anim. Behav..

[44] Galván I, Jorge A, Nielsen JT, Møller AP (2019). Pheomelanin synthesis varies with protein food abundance in developing goshawks. J. Comp. Physiol. B.

[45] Zaragoza-Trello C, Vilà M, Botías C, Bartomeus I (2021). Interactions among global change pressures act in a non-additive way on bumblebee individuals and colonies. Funct. Ecol..

[46] Rollin, N. A note on abnormally marked Song Thrushes and Blackbirds. *Trans. Nat. Hist. Soc. Northumberl. Durh. Newctle upon Tyne* **10**, 183–184 (1953).

[47] Guerrero-Bosagna, C. *et al.* Transgenerational epigenetic inheritance in birds. *Environ. Epigenet.* **4**, dvy008 (2018). 10.1093/eep/dvy008 PMC5920295 29732172

[48] Camacho C, Negro JJ, Redondo I, Palacios S, Sáez-Gómez P (2019). Correlates of individual variation in the porphyrin-based fluorescence of red-necked nightjars (*Caprimulgus ruficollis*). Sci. Rep..

[49] Camacho C (2013). Tropical phenology in temperate regions: extended breeding season in a long-distance migrant. Condor.

[50] Cleere N (2010). Nightjars: a guide to nightjars and related birds.

[51] Gargallo G (1994). Flight feather moult in the red-necked nightjar *Caprimulgus ruficollis*. J. Avian Biol..

[52] Jackson HD (2003). A field survey to investigate why nightjars frequent roads at night. Ostrich.

[53] Jackson HD (1984). Finding and trapping nightjars. Bokmakierie.

[54] Senar JC, Pascual J (1997). Keel and tarsus length may provide a good predictor of avian body size. Ardea.

[55] Svensson L (1992). Identification Guide To European Passerines.

[56] van de Pol M, Wright J (2009). A simple method for distinguishing within-versus between-subject effects using mixed models. Anim. Behav..

[57] Schielzeth H, Forstmeier W (2009). Conclusions beyond support: overconfident estimates in mixed models. Behav. Ecol..

[58] Rising JD, Somers KM (1989). The measurement of overall body size in birds. Auk.

[59] Magnusson, A. *et al.* Package “glmmTMB”. R Package Version 0.2.0. (2017).

[60] Hartig, F. DHARMa: residual diagnostics for hierarchical (multi-level/mixed) regression models. R package version 0.2, 4. (2019).

[61] Nakagawa S, Schielzeth H (2013). A general and simple method for obtaining R2 from generalized linear mixed-effects models. Methods Ecol. Evol..

[62] Barton, K. MuMIn: Multi-Model inference. Model selection and model averaging based on information criteria (AICc and alike). R package version 1.43.17. (2020).

